# Landscape context and substrate characteristics shape fungal communities of dead spruce in urban and semi‐natural forests

**DOI:** 10.1111/1462-2920.15903

**Published:** 2022-01-26

**Authors:** Aku Korhonen, Otto Miettinen, Johan D. Kotze, Leena Hamberg

**Affiliations:** ^1^ Natural Resources Institute Finland (Luke) Helsinki Finland; ^2^ Finnish Museum of Natural History University of Helsinki Helsinki Finland; ^3^ Ecosystems and Environment Research Programme, Faculty of Biological and Environmental Sciences University of Helsinki Helsinki Finland

## Abstract

Urban green areas are becoming increasingly recognized for their biodiversity potential. However, little is known about how urbanization shapes cryptic species communities, such as those residing in deadwood. In this study, we investigated downed Norway spruce trunks at intermediate stages of decay, in urban and semi‐natural forests in southern Finland. To understand the interconnections between landscape context, deadwood characteristics and wood‐inhabiting fungal communities, we studied structural characteristics, surface epiphyte cover and internal moisture and temperature conditions of the tree trunks, and fungal communities residing in the wood. Our findings showed that urban tree trunks had less epiphyte cover and lower moisture than trunks in semi‐natural forests. Overall, urban forests provide less favourable habitats for a majority of the dominant wood‐inhabiting fungal species and for red‐listed species as a group. Yet, 33% of urban trunks hosted at least one red‐listed species. While these landscape‐scale effects may be driven by local climatic conditions as well as contingencies related to available species pools, our results also highlight the significance of substrate‐scale variability of deadwood in shaping wood‐inhabiting fungal communities. We show that epiphyte cover is a significant driver or indicator of these small‐scale dynamic processes in deadwood.

## Introduction

Intensive forestry use has drastically reduced the supply of deadwood in northern European forests, which has led to a reduction in the abundance and diversity of deadwood dependent organisms (Siitonen, 2001). Significant increases in dead‐wood retention are difficult to reconcile in the production forestry sector, but in multi‐use forests such as forested urban green spaces and recreational forests, biodiversity‐oriented management can be easier to accommodate (Gundersen *et al*., 2005). The restoration of ecologically important habitat structures, such as deadwood, could be viewed as an opportunity to offset habitat loss due to urbanization (Le Roux *et al*., 2014). On the other hand, the ecological value of urban forests is lowered by habitat fragmentation and other environmental disturbances associated with human activity (Rebele, 1994; Grimm *et al*., 2008). These negative impacts constrain the range of forest specialist species that urbanized environments can support (Ramalho *et al*., 2014; Piano *et al*., 2020). Therefore, to assess the potential of urban forests for biodiversity conservation and to inform management of habitat resources in urbanized areas, the effects of urbanization on species need to be investigated.

Here, we focus on deadwood which has been, until recently, little studied in the urban milieu (e.g. Korhonen *et al*., 2021; Meyer *et al*., 2021). Decaying wood forms dynamic microhabitats and food sources that sustain approximately 20%–25% of forest species in boreal Fennoscandia (Siitonen, 2001), and wood‐inhabiting fungi (WIF) are key drivers in the wood‐decay process (Stokland *et al*., 2012). In northern European forests, decaying Norway spruce [*Picea abies* (L.) H. Karst.] logs are among the most species‐rich dead‐wood types and the preferred substrate for many threatened specialist WIF species (Junninen and Komonen, 2011; Huuskonen *et al*., 2021). Spruce logs are also one of the main commodities of the wood industry, and therefore, only a small fraction is incorporated into the deadwood reservoirs in managed forest stands. A recent study on spruce‐inhabiting polypore diversity along an urban–rural gradient (Korhonen *et al*., 2021) found that coarse woody debris in urban forests can host red‐listed species. However, their frequency in urbanized areas was still lower than expected after accounting for deadwood quantity and local‐scale forest connectivity. To what extent this trend was due to adverse environmental conditions versus larger‐scale habitat availability remains unresolved.

**Fig. 1:**
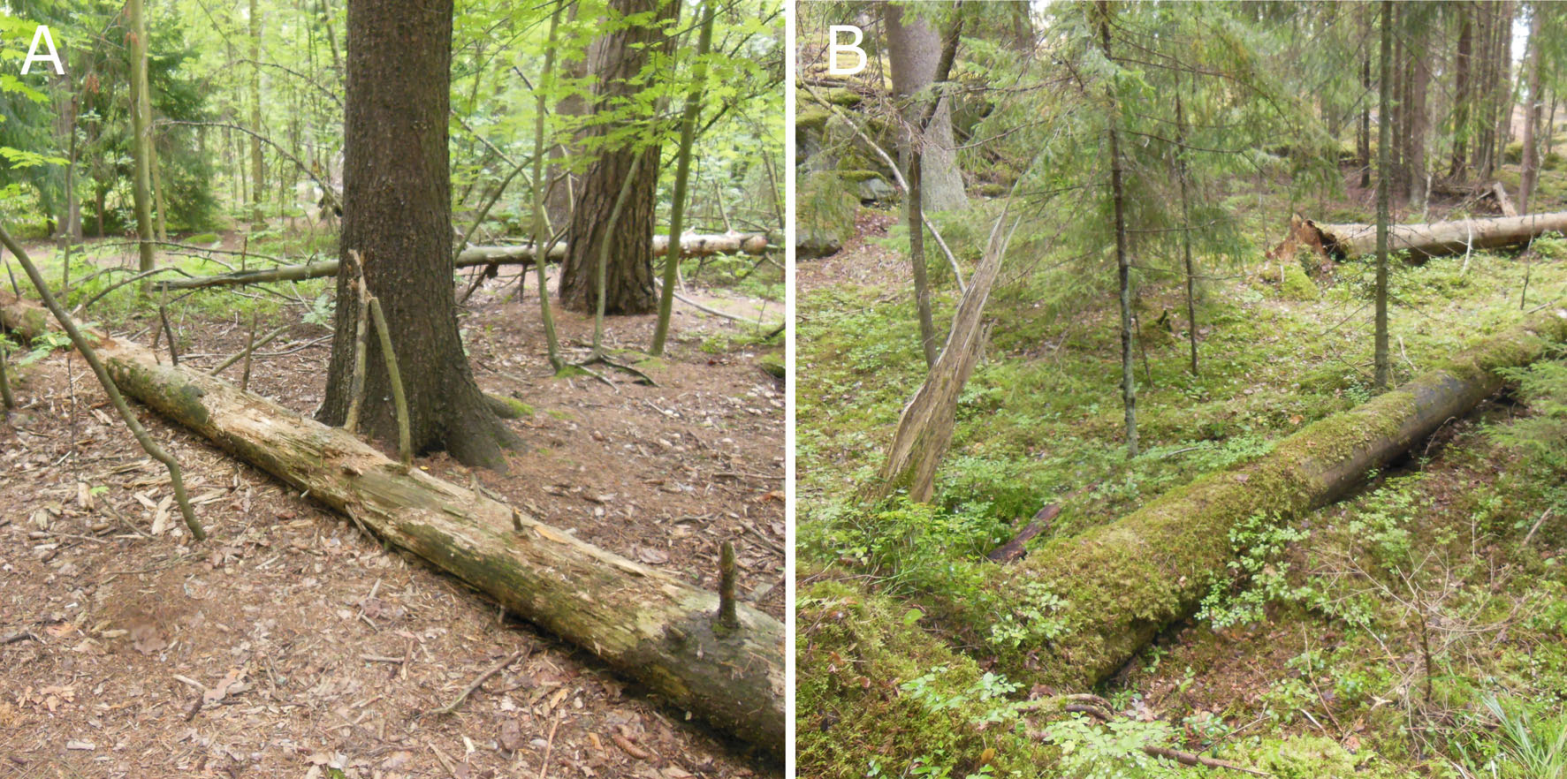
Heavily worn (A) and mostly intact (B) decaying Norway spruce (*Picea abies*) trunks in mesic heath forests in Helsinki, Finland. The forest stand in panel A is situated in a remnant forest area within the urban matrix (approximately 4000 residents km^−2^). Intensive trampling has eroded the surface layer of the decaying tree trunk and eliminated most of the vegetation on the forest floor. The forest stand in panel B is situated in a recreational forest area at the urban fringe (<200 residents km^−2^). Forest vegetation at this site is mostly intact and epiphytic vegetation has developed on the surface of the decaying tree trunk. Both trunks were included in the present study. Photos: Aku Korhonen.

Earlier, fruiting‐body based studies have indicated microclimatic effects on WIF (Pouska *et al*., 2016), with low moisture and high temperature fluctuations being associated with lower WIF species richness (Pouska *et al*., 2017). These effects are expected to be pronounced in urban forests that are often highly fragmented (Liu *et al*., 2016) with high proportion of edge habitat. Furthermore, downed deadwood on the forest floor is exposed to direct human disturbances such as trampling. In places where recreational use is intense, mechanical wear from trampling can prevent the development of epiphyte cover on downed deadwood, eventually eroding the decaying wood itself (Fig. 1).

In this study, we investigated how urbanization affects the properties of dead wood and WIF communities. We studied 90 decaying spruce trunks distributed in urban forests and semi‐natural rural forests in the Greater Helsinki area, southern Finland. WIF communities were analyzed with ITS2 metabarcoding, allowing us to identify species present in the wood in vegetative states. We hypothesized that wood temperature and moisture conditions are important factors shaping WIF community in decaying spruce trunks, and that urban trunks are prone to desiccation and more fluctuating temperatures due to edge effects. Furthermore, we expected that reduced epiphyte cover in urban forests, due to the wear of tree trunks, could accentuate these characteristics (Chang *et al*., 2019). More specifically, we wanted to test whether these changes affect the diversity of WIF communities and the occurrence of WIF species of conservation concern.

## Results

### Fungal diversity

Downed intermediately decayed spruce trunks and their properties were studied in 24 urban forest sites (66 trunks) and eight semi‐natural forest sites (24 trunks). Altogether, 475 operational taxonomic units (OTUs), identified as fungi, were detected (with minimum representation of 5‰ of total sequences in at least one sample) within the 360 sequenced samples (four per trunk). Twelve OTUs represented red‐listed WIF species (Table 1).

**Table 1:** Red‐listed WIF species detected in downed spruce trunks in urban and semi‐natural forests.

Species	Red‐list status^a^	Observations in urban forests (*n* = 66)	Observations in semi‐natural forests (*n* = 24)
*Amylocorticium subincarnatum*	VU_FI_, EN_NO,SE_	1	0
*Crustoderma dryinum*	NT_FI_, VU_NO,SE_	1	2
*Fomitopsis rosea*	NT_EE,FI,NO,SE_	0	1
*Phellinidium ferrugineofuscum*	NT_EE,SE_	2	0
*Phellopilus nigrolimitatus*	NT_NO,SE_	6	2
*Phlebia centrifuga*	VU_SE_	0	3
*Phlebia subulata*	VU_NO,SE_	6	4
*Postia undosa*	NT_LE,NO_, VU_SE_	1	1
*Pycnoporellus fulgens*	EN_NO_	5	1
*Rhodonia placenta*	VU_SE_, EN_NO_	2	2
*Skeletocutis brevispora*	NT_FI_, VU_NO,SE_, CR_EE_	0	1
*Skeletocutis delicata*	NT_FI_	1	0
Proportion of trunks where red‐list species were detected		33%	54%

Species‐specific frequencies are given as the number of tree trunks where the species was detected. Proportions (%) of tree trunks where red‐listed species were detected refer to trunks with at least one red‐listed species. Red List status of each species is indicated based on the national or regional red list assessments from boreal north‐eastern Europe. Taxonomic nomenclature according to the UNITE database v8.2.

Region: EE = Estonia, FI = Finland, LE = Leningrad Region (Russia), NO = Norway, SE = Sweden.

^a^ Status: NT = near threatened, VU = vulnerable, EN = endangered, CR = critically endangered.

### Relationship between WIF community composition and tree trunk variables

**Fig. 2:**
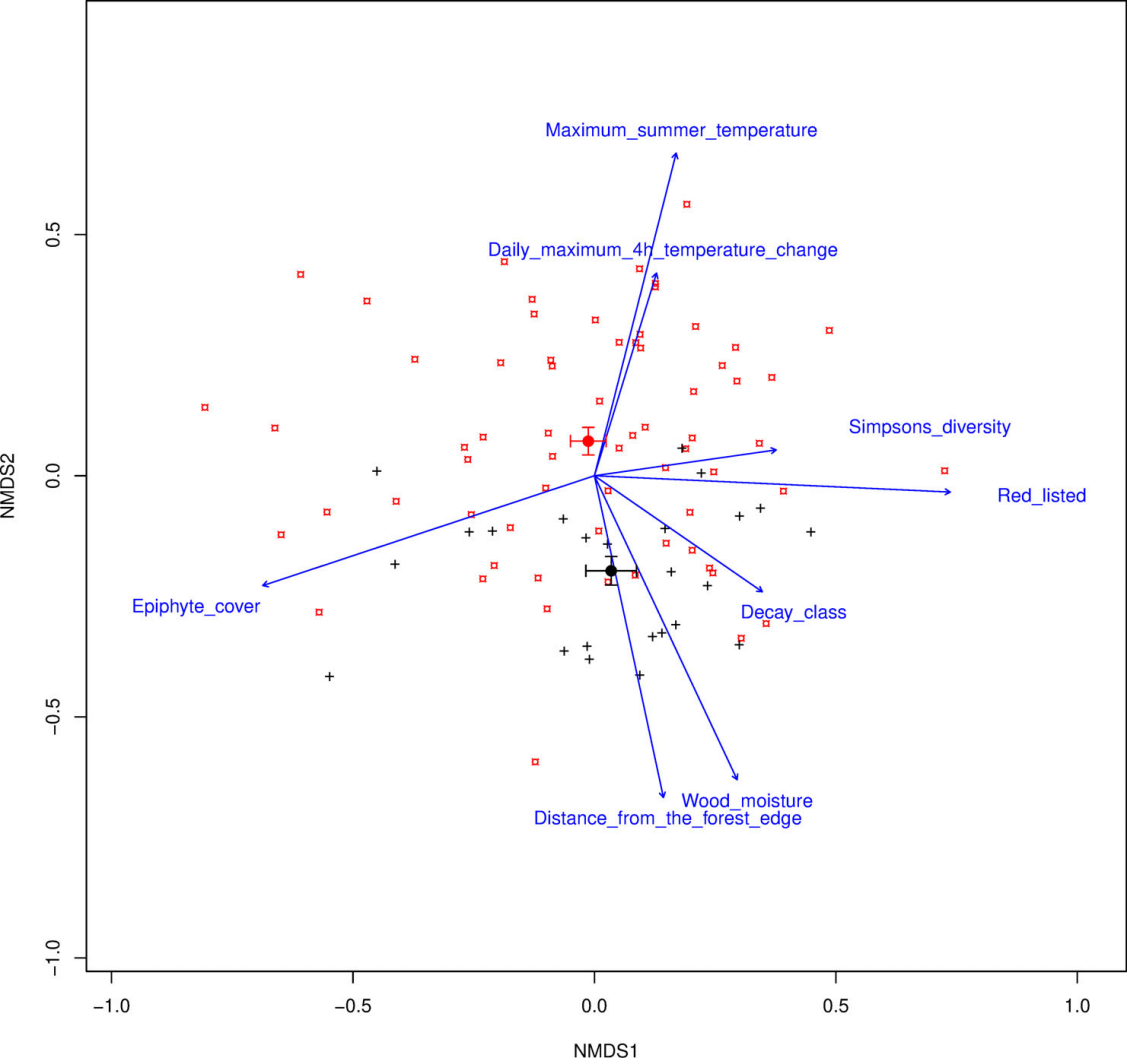
Two‐dimensional projection of the 3D‐NMDS ordination of 90 downed spruce trunks based on WIF community composition. Points depict the positions of individual spruce trunks from urban (red ¤) and semi‐natural (black +) forests in ordination space. Centroids (±SE) of urban and semi‐natural tree trunks are denoted with solid red and black circles. Variable correlations are depicted as vectors with their length proportional to the strength of the relationship (*R* ^2^). Only variables with significance level *p* < 0.05, based on permutation test (permutations = 10 000), are shown. See Supplementary Table B1 for *R* ^2^ and *p*‐values for all fitted variables and Supplementary Table C2 for intercorrelations between variables.

Non‐metric multidimensional scaling (NMDS) of the WIF community data (*n* = 90; stress = 0.186) showed that variation in community composition was ordered along two approximately perpendicular environmental gradients (Fig. 2). One gradient was related to the amount of epiphyte cover on the deadwood substrate while the other was related to distance from the forest edge, wood moisture and temperature conditions inside the wood. Simpson's diversity index (SDI) of the WIF community and the number of red‐listed species were aligned with the gradient related to epiphyte cover, both increasing with decreasing epiphyte cover. Differentiation in community composition between tree trunks in urban and semi‐natural rural forests followed the gradient involving distance from the forest edge, wood moisture and temperature conditions. However, variation in community composition was overall highly overlapping between urban and semi‐natural rural forests. The full statistical output of the variable fitting of the NMDS‐ordination is presented in Supplementary Table B1.

### Linkages between landscape setting, tree trunk variables and the WIF community

**Fig. 3:**
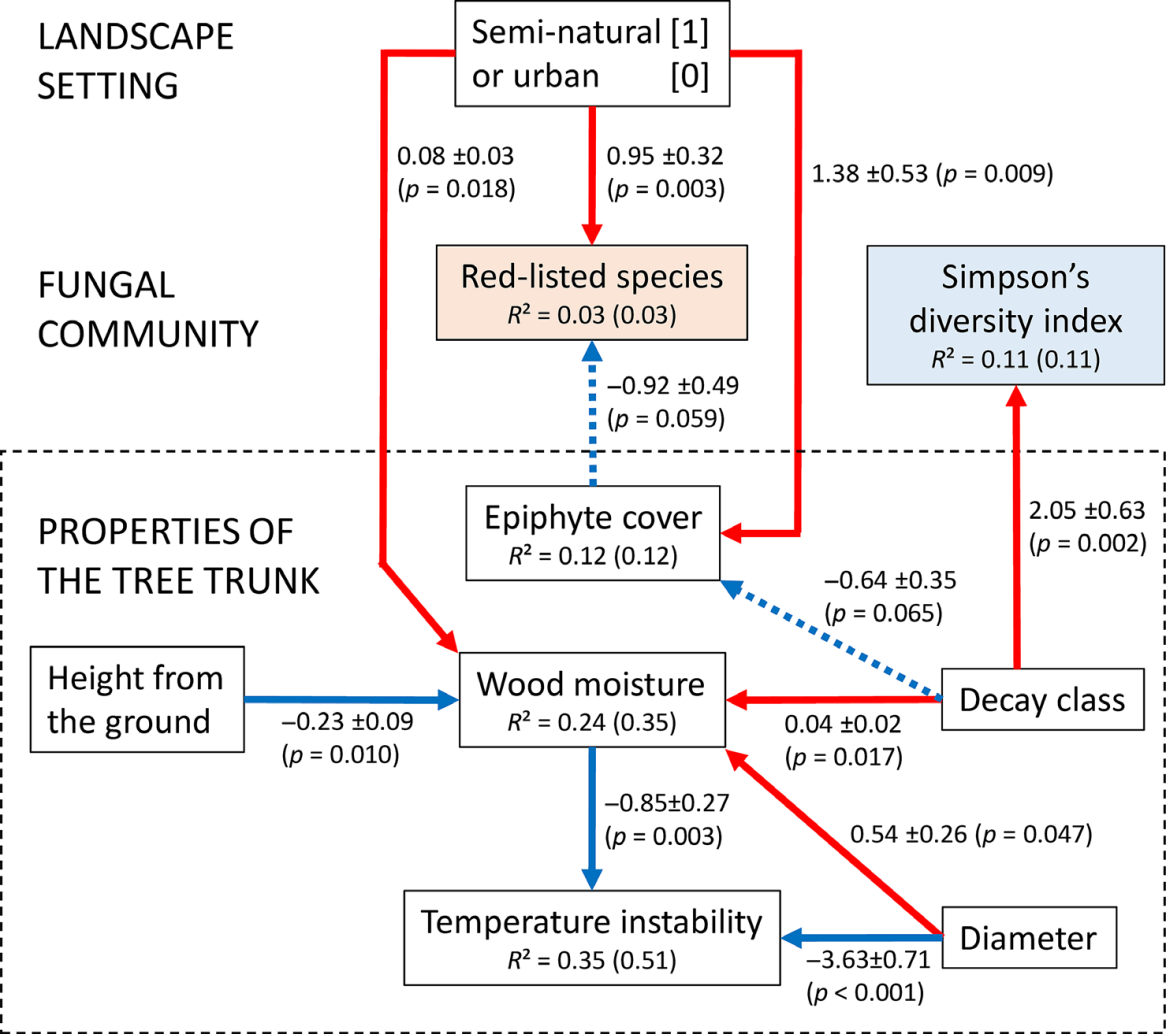
Refined path model depicting interconnections between landscape setting, tree trunk characteristics and WIF community structure (*n* = 90). Solid arrows depict paths at significance level *p* < 0.05 and dashed arrows depict paths at significance level 0.05 ≤ *p* < 0.10. Red colour indicates a positive effect and blue a negative effect. Associated path coefficients (unstandardized estimate ± SE) are presented next to the arrows. Coefficients of determination (*R* ^2^) are given for dependent variables. Both marginal values (variance explained by fixed effects only) and conditional values (variance explained by fixed and random effects) are presented, the latter in parentheses.

Decaying tree trunks in urban forests were drier and had less epiphyte cover than trunks in semi‐natural forests (Fig. 3). Wood moisture further modulated temperature instability inside the tree trunk (daily maximum 4 h temperature change), together with trunk diameter. Neither wood moisture nor temperature instability was significantly associated with epiphyte cover, but epiphyte cover was negatively associated with the number of red‐listed species present in a trunk. The overall diversity (SDI) of the WIF community was associated with decay class only, increasing with advancing decay.

The refined path model (Fig. 3) was consistent with the data (Fisher's *C* = 32.05, df = 38, *p* = 0.74). Detailed model output, including *p* values for path coefficients and tests of directed separation underlying the path analysis are presented in Supplementary Table B2.

### Species niches in relation to environmental variables

Species niches of some of the most common and abundant OTUs were estimated by fitting a joint species distribution model based on ITS2 sequence abundance. Based on standardized model coefficients, landscape setting, i.e. whether the forest site was urban or semi‐natural (Naturalness), was generally the most influential variable explaining the abundance of individual OTUs in wood samples (Table 2). Most OTUs responded positively to the semi‐natural forest environment and only few preferred the urban setting. In terms of epiphyte cover, most OTUs showed significantly negative responses to increasing epiphyte cover, and only few responded positively. Niche specialization in terms of decay class and trunk diameter varied among OTUs.

**Table 2:** Results from the joint species distribution model.

OTU	Lifestyle	Frequency	Sequence abundance	SR^2^	Model coefficients						
					Intercept	Sequencing depth	Diameter	Decay class 2	Decay class 4	Epiphyte cover	Naturalness
*Talaromyces rademirici*	Unspecified saprotroph	25	44k	0.25	−10.84	2.98	−0.12	−0.13	1.21	−2.38	−0.13
*Ascocoryne cylichnium*	Wood saprotroph	44	201k	0.39	−13.43	3.04	0.03	−0.65	−0.77	0.67	2.70
*Meliniomyces* OTU0003	Root endophyte/soil saprotroph	77	318k	0.56	−10.30	3.44	−0.03	−0.92	1.14	−1.34	1.41
Helotiales OTU0016	Unknown	17	52k	0.27	−14.60	3.07	0.01	0.72	−1.27	−0.99	−1.06
*Peniophorella praetermissa*	Wood saprotroph: white‐rot	33	103k	0.05	−11.62	2.60	−0.02	0.08	−0.43	−0.64	0.13
*Resinicium bicolor*	Wood saprotroph: white‐rot	21	281k	0.07	−12.38	1.94	0.02	0.02	−0.67	1.18	1.34
*Alutaceodontia alutacea*	Wood saprotroph: white‐rot	21	46k	0.71	−12.15	2.01	0.06	−0.53	−0.41	0.19	2.64
*Antrodia serialis*	Wood saprotroph: brown‐rot	27	394k	0.50	−13.19	3.42	−0.06	−0.33	−0.34	−0.80	0.68
*Fomitopsis pinicola*	Wood saprotroph: brown‐rot	41	982k	0.43	−10.38	3.29	−0.08	−0.65	0.32	−1.14	0.89
*Phlebia livida*	Wood saprotroph: white‐rot	29	254k	0.66	−14.06	2.04	0.14	−0.48	−0.63	0.30	2.38
*Heterobasidion parviporum*	Plant pathogen/wood saprotroph: white‐rot	40	932k	0.60	−10.77	1.46	0.20	0.95	−1.88	2.81	−1.80
*Helicogloea dryina*	Wood saprotroph	32	177k	0.25	−32.07	5.51	0.12	−1.30	0.13	−0.14	4.56
*Dacrymyces stillatus*	Wood saprotroph: brown‐rot	29	162k	0.07	−10.23	2.75	−0.10	0.58	−0.56	−0.99	−1.12
Basidiomycota OTU0014	Unknown	31	61k	0.91	−16.29	3.06	0.05	−0.81	0.82	−1.72	2.72

Information about OTU lifestyle is based on Põlme *et al*. (2020).

Frequency of OTUs is presented as the number of tree trunks where the OTU was present in at least one sample with minimum 5‰ of the total sequence reads. Explanatory power of the joint species distribution model is expressed as *SR* ^*2*^. Species niches are expressed with standardized model coefficients (posterior means). Red colour indicates a positive and blue indicates a negative effect of an explanatory variable on the abundance (read count) of an OTU. Darker colours indicate 95% posterior probability and lighter colours indicate 90% posterior probability for coefficients deviating from 0 (white cells indicate non‐significant effects).

Mean explanatory power (*SR* ^2^) of the joint species distribution model averaged across all 14 OTUs was 0.41 (0.05–0.91). For most OTUs, more variance was explained by random effects representing unmeasured processes and environmental variability than by fixed effects representing measured variability. OTU‐specific variance partitioning is depicted in Supplementary Fig. B1.

## Discussion

### Decaying spruce trunks as WIF habitats in urban and semi‐natural settings

According to our observations, decaying spruce trunks in urban forests were drier and had less epiphyte cover than corresponding substrates in rural semi‐natural forests. These environmental gradients are consistent with the increasing degree of forest fragmentation from rural to urban landscapes (Liu *et al*., 2016). Due to small forest fragment size and the proximity of forest edges (Supplementary Table C1), urban deadwood is exposed to increased temperature variability (Hofmeister *et al*., 2019) and reduced humidity (Malmivaara‐Lämsä *et al*., 2008a; Crockatt and Bebber, 2015) relative to buffered interior forest conditions. Lower epiphyte cover on urban deadwood may also be partly explained by urban climatic conditions (Wiklund and Rydin, 2004; Stewart and Mallik, 2006). In addition to edge effects, the loss of field and ground layer vegetation (Supplementary Table C2) due to heavy recreational use can make the local environment of downed deadwood more exposed and less humid. Epiphytic vegetation growing on downed wood is further exposed to direct mechanical wear caused by trampling.

Our results did not reveal significant associations between epiphyte cover and wood moisture‐temperature conditions, but both environmental gradients were significantly associated with WIF community composition. Wood moisture‐temperature gradient appeared to be more involved with the differentiation between urban and semi‐natural WIF assemblages than epiphyte cover, although variation in community composition overlapped considerably between the two landscape settings. Our results suggest that WIF communities are sensitive to an altered urban climate, but the true mechanisms may also involve other unmeasured variability. For instance, managed and urbanized forest landscapes provide relatively low density of large‐diameter deadwood compared to semi‐natural settings (Lehvävirta, 1999; Korhonen *et al*., 2020), and thus, urban deadwood may be exposed to less abundant and less diverse WIF inoculum from ambient spore rain (Abrego *et al*., 2020). This effect probably explains the lower average occurrence of red‐listed WIF species in urban spruce trunks. Many of these species appear to be relatively specialized in their resource use, gaining competitive advantage over generalist WIF species only when suitable deadwood resources are abundant enough in the landscape (see e.g., Nordén *et al*., 2013). However, our results provide no evidence to suggest that red‐listed WIF species would be further hindered by the altered quality of deadwood in urban forests, i.e. reduced wood moisture or epiphyte cover. On the contrary, red‐listed species tended to be more numerous in trunks that had sparse epiphyte cover.

The overall diversity (SDI) of WIF communities at the trunk level did not differ significantly between trunks in urban and semi‐natural settings, but our results suggest differences in the identity of the dominant WIF components of the communities. As most of the dominant WIF species were more abundant in semi‐natural settings, the dominance of urban WIF communities was distributed to a narrower set of species. Notably, the most prominent urban‐associated WIF species was *Heterobasidion parviporum* which is an aggressive pathogen of spruce and is capable of infecting living trees vegetatively through root connections (Piri, 1996). This species benefits from root disturbances as well as soil eutrophication (Gaitnieks *et al*., 2021), which are common processes in urban forests (Lovett *et al*., 2000; Hamberg *et al*., 2009; O'Brien *et al*., 2012).

### Significance of epiphyte cover on decaying spruce trunks

The cover of epiphytes on decaying tree trunks is a dynamic property that develops along the decay process (Kushnevskaya *et al*., 2007). Because the successional changes occurring within and on the surface of deadwood are parallel, the specific effects of epixylic vegetation succession can be difficult to disentangle in observational studies involving a wide range of deadwood qualities (e.g. Pouska *et al*., 2011, 2016, 2017; Ruokolainen *et al*., 2018). Our results, focusing on a specific deadwood quality and restricted decay stage range, provide support for a distinct association between wood epiphyte cover and WIF community composition.

Although our results failed to identify significant linkages between epiphyte cover and wood moisture‐temperature conditions, earlier experimental work has shown that the removal of epiphyte cover increases seasonal fluctuations in wood moisture content (Chang *et al*., 2019). Thus, temporal dynamics in moisture conditions, which could not be captured by our measurements, could represent a plausible mechanism for the association between epiphyte cover and the WIF community. Bryophyte mats have also been suggested to insulate deadwood from fungal spore rain (Dynesius *et al*., 2010). Under blanketed conditions, competitive interactions between pre‐existing WIF species could then result in the dominance of the most competitive species and a reduction in community diversity (Toljander *et al*., 2006), which would be consistent with the observed trend of lower SDI in highly epiphyte‐covered trunks. Reciprocally, exposed wood surfaces could maintain diversity by allowing the continued introduction of fungal species from ambient spore rain. Negative effect on fungal richness could also result from antifungal compounds leaching from bryophyte mats (Frahm, 2004) into the decaying wood.

Only few of the specifically analyzed OTUs showed preference for epiphyte blanketed tree trunks. Among them were two white‐rotting basidiomycetes *Heterobasidion parviporum* and *Resinicium bicolor* and one wood‐saprotrophic ascomycete *Ascocoryne cylichnium* that is also known to occur as a tree endophyte (Vasiliauskas and Stenlid, 1998). OTUs responding negatively to epiphyte cover were more numerous than positively responding OTUs and included all three brown‐rotting species that were included in the analysis: *Fomitopsis pinicola*, *Antrodia serialis* and *Dacrymyces stillatus*. All of the species mentioned above are early colonizers of deadwood (Ovaskainen *et al*., 2013) and usually establish before epiphytes grow over the wood surface. Therefore, their affinities with epiphyte cover seem to reflect niche specialization or competitiveness, rather than their ability to colonize the substrate. We cannot rule out a possible bottom‐up effect from the fungal community to the epiphytic vegetation, e.g. through the physicochemical properties of the decaying wood determined by wood‐decaying fungal species (Fukasawa *et al*., 2015). However, we presume that this effect would be more relevant in terms of epiphyte species composition (cf. Ando *et al*., 2017) and less so for the total coverage of epiphytes.

Although the reduction of epiphyte cover had putatively beneficial effects on many fungal species, the effects of mechanical wear in urban forests would still need to be studied more closely. Erosion of the tree trunk probably accelerates decomposition and could interfere with fungal fruiting‐body formation. Most vulnerable species would be expected to be those that fruit predominantly in later decay stages (see Renvall, 1995) when the substrate is most susceptible to mechanical destruction.

### Conclusions

Our results suggest that WIF communities of decaying spruce trunks in urban forests are equally diverse as those in semi‐natural forest settings at the substrate level, but that species composition in urban deadwood is slightly shifted. Responses of WIF communities to the urban forest environment may reflect environmental effects, such as greater susceptibility to wood desiccation, but larger‐scale differences in available WIF species pools could also contribute to this pattern. Some WIF species, especially threatened large‐diameter deadwood specialists, are probably dispersal‐limited in managed and fragmented urban forest landscapes where the retention of suitable deadwood substrates has been restricted. Yet, threatened species are not excluded from urban forests and we detected them in 33% of urban spruce trunks. Our results also highlight the potential role of epiphyte cover on vegetative WIF assemblages in decaying spruce and reveal contrasting responses to this substrate characteristic among some common spruce‐associated WIF species. The extent of epiphyte cover on intermediately decayed spruce trunks was variable also in semi‐natural forests, but lower average cover on urban trunks is indictive of retardation or disruption of natural epixylic vegetation succession in urbanized forest environments. Further studies would be needed to pinpoint causal mechanisms underlying the associations between WIF communities and epiphytes.

## Experimental procedures

### Selection of forest sites and spruce trunks

All study sites represented forest stands with natural herb‐rich to mesic heathland forest vegetation, Norway spruce as the dominant tree species, and the age of dominant living trees at least 60 years. Urban study sites (24 sites, 66 trunks) were distributed across the Helsinki metropolitan area (combined population of approximately 1.2 million; Statistics Finland, 2020) in southern Finland, and semi‐natural study sites (eight sites, 24 trunks) in the surrounding rural areas (Supplementary Fig. C1). Urban sites represented deadwood hotspots within the urban landscape where deadwood had accumulated in substantial volumes locally. Semi‐natural sites represented unmanaged stands with large volumes of deadwood and minimal signs of logging history and other human disturbances, including recreational use.

At each site, we searched for fallen Norway spruce trunks that were at intermediate stages of decay [predominantly decay class 3 according to Renvall's, 1995 five‐stage classification] and had a diameter of 20–40 cm at 4–6 m from the base. Decay class 3 is defined as the stage in which the decaying trunk has already partly rotten (knife penetrates approximately 3–5 cm into the wood easily) but still retains its shape. At this decay stage, spruce trunks are mostly decorticated, and the wood surface is being colonized by epixylic lichens and bryophytes. In the urban forest sites, all suitable tree trunks (1–4 trunks per site) were included. In semi‐natural areas, we chose three suitable trunks per site randomly.

All trunks were situated inside a closed forest with mean canopy openness of 23% (SD = 5). Distance between the trunk and the nearest forest edge varied from 7 to 589 m (Supplementary Table C1). Trunks in urban forests were generally closer to forest edges (median 53 m) than trunks in semi‐natural forests (median 216 m). However, neither canopy openness nor distance to the forest edge had any significant associations with properties of the tree trunks (Supplementary Table C2).

### Measurement of tree trunk variables

Characteristics of the downed spruce trunks were measured in June 2019. Measurements were taken from four 2 m segments between 1 and 9 m from the base of the tree trunk (Supplementary Fig. A1). Trunk diameter and distance between the bottom of the trunk and the soil surface directly below it was measured at the midpoint of each segment. Decay class (1–5) was determined for each segment based on knife penetration into the wood. Epiphyte cover (%) was measured for the top surface of each segment. Epiphyte cover consisted primarily of bryophyte mats. Wood moisture content (%), based on electric resistance, was measured (Moisture Meter ET‐928, Clas Ohlson, Insjön, SE) from the shadier vertical side of the tree trunk at the midpoint of each segment. Measurements were made on an intact wood surface exposed by removing epiphytes, remains of bark and the most decayed layer of wood from the surface. The month of June, when all measurements were made, is at the end of the driest period of the year (Finnish Meteorological Institute, 2021) when wood moisture was expected to be close to the annual minimum.

Temperature inside each tree trunk was monitored between the spring and fall of 2019 with a datalogger (iButton Thermochron DS1921G‐F5, Maxim Integrated, San Jose, CA, USA) embedded in the middle of the trunk. Dataloggers were inserted into drilled holes that were sealed tightly with wooden plugs. Temperature measurements were recorded synchronously with 4 h intervals from 1 AM to 9 PM. To get an indication of how sensitive temperature inside the tree trunk was to external temperature changes, we extracted the maximum 4 h temperature change for each day of the measurement period between 1 May and 29 September. These values were then averaged across the days to yield one value for each trunk, the mean of daily maximum 4 h temperature changes.

### DNA sampling and fungal community analysis

Wood samples were collected from spruce trunks in October 2018. Four wood samples were taken from each tree trunk at 2, 4, 6 and 8 m distance from the base (Supplementary Fig. A1). Samples were extracted from the shadier side of the trunk. Epiphytes, bark and loose rotten wood were removed with a flame‐sterilized knife to expose a clean surface of solid wood on the side of the trunk. Wood shavings were extracted with a flame‐sterilized 6 mm diameter drill and collected into paper bags and stored at −20°C until DNA extraction.

DNA was extracted with a NucleoSpin Soil (Macherey‐Nagel, Düren, DE) extraction kit from 112 ± 52 mg (mean ± SD) of sample material. Extraction was done according to the manufacturer's instructions with lysis buffer SL2 and final elution in 30 μl volume. The ITS2 region was amplified using PCR with primers gITS7 (Ihrmark *et al*., 2012) and ITS4 (White *et al*., 1990) with 6 bp dual index for 28 cycles. The resulting PCR fragments were sequenced with the MiSeq v3 (Illumina, San Diego, CA, USA) 2 × 300 bp paired‐end system yielding 20–25 M raw sequence reads.

Quality filtering and the removal of artefacts, primer‐dimers and primers from raw sequence reads were conducted with the PipeCraft 1.0 pipeline (Anslan *et al*., 2017). A detailed description of the analysis steps from sequence quality filtering to the clustering of sequences into OTUs and taxonomic annotations is provided in Supplement D.

The diversity of fungal communities was measured with the SDI. Before calculating the index, relative OTU abundances (read count divided by sample sequencing depth) were averaged between the two middlemost samples for each trunk (segments 2 and 3; Supplementary Fig. A1) to aggregate the data to trunk‐level and to focus on the data that were most closely connected to the temperature measurement point within the tree trunk. Averaged OTU abundances were then used for calculating the index with R package *iNEXT* v.2.0.20 (Chao *et al*., 2014; Hsieh *et al*., 2020).

To assess the value of tree trunks as habitat for WIF species of conservation concern, we recorded the number of OTUs representing red‐listed species present in each individual trunk. We considered species included in the national or regional IUCN Red List assessments across the hemi‐ and southern boreal north‐eastern Europe, i.e. Estonia (see Runnel *et al*., 2021), Finland (Kotiranta *et al*., 2019), Norway (NBIC, 2019), Sweden (SLU Artdatabanken, 2020) and the Leningrad Region of Russian Federation (Geltman *et al*., 2018). A species was recorded as present if the corresponding OTU accounted for at least 5‰ of the total sequence reads in at least one sample.

### Relationships between WIF community composition and environmental variables

To analyze how environmental and tree trunk variables were related to wood‐inhabiting fungal community composition, we applied NMDS to produce an ordination onto which variables were fitted. A three‐dimensional ordination solution was produced based on a dissimilarity matrix calculated using the Raup–Crick distance (calculated with function ‘*vegdist*’) based on presence–absence data of OTUs using the R package *vegan* v.2.5‐6 (Oksanen *et al*., 2019). Optimal ordination was searched by running the analysis 50 times and choosing the solution with the lowest stress value. The analysis was performed at the tree trunks level (*n* = 90), focusing on the middle 4 m of each trunk closest to the temperature measurement point. Therefore, segment‐specific measurements were aggregated to trunk‐level by averaging measurements between the two middlemost trunk segments (Supplementary Fig. A1). OTUs were considered present if their read counts accounted for at least 5‰ of the total sequence reads in either of the two samples.

### Linkages between landscape setting, tree trunk variables and WIF community

To test our hypotheses about the interconnections between different tree trunk variables and to explore their relevance for fungal diversity and the occurrence of red‐listed species, we used path analysis implemented with the R package *piecewiseSEM* v.2.1.0 (Lefcheck, 2016). First, we defined a preliminary path model representing the hypothesized mechanistic structure linking wood moisture and temperature instability to structural characteristics of the tree trunk. Wood moisture was expected to be increased by epiphyte cover (insulation), diameter and decay class (water holding capacity) and reduced by height from the ground (detachment from soil moisture). Temperature instability inside the tree trunk was expected to be reduced by epiphyte cover and trunk diameter (insulation), wood moisture (inertia against temperature fluctuations) and increased by height from the ground (increased exposure to air temperature fluctuation). Furthermore, we expected that deadwood in urban forests would be covered less by epiphytes due to edge effects and trampling‐induced erosion of the vegetation (Malmivaara‐Lämsä *et al*., 2008b), and that red‐listed species would be more common in semi‐natural forests due to longer historical habitat continuity (Penttilä *et al*., 2004; Berglund *et al*., 2011; Nordén *et al*., 2018). Diversity of fungal community was expected to increase with advancing decay stage (Rajala *et al*., 2012).

We used the same set of data as in the ordination analysis (*n* = 90). Wood moisture, temperature instability (mean of daily maximum 4 h temperature changes) and SDI were modelled with linear mixed models following a normal distribution. Epiphyte cover was modelled with a generalized linear mixed model following a binomial distribution with a logit link function. The number of red‐listed species detected in a trunk was modelled with a generalized linear mixed model following Poisson distribution with a log link function. Forest site was included as a random factor in the models.

After fitting the initial path model, non‐significant links were removed and new links were added if their inclusion was supported by a significant *p*‐value indicating deviation from expected conditional independence assessed using Shipley's (2000) d‐separation test. Statistical support for the refined model structure was assessed from Fisher's *χ* ^2^ distribution *C* statistic comparing observed correlations across independence claims to random variation (Shipley, 2009).

### Species niches in relation to tree trunk variables and landscape context

To examine how species respond to their environment, we fitted joint species distribution models with Hierarchical Modelling of Species Communities (HMSC) implemented in the R‐package *HMSC* v.3.0‐6 (Tikhonov *et al*., 2020). We included OTUs that represented at least 12% of the total sequence reads in at least one sample and were present in at least 25 samples with a minimum representation of 5‰ of the total sequence reads within the sample. We used data from every sampled trunk segment individually (*n* = 360).

We used read counts of the OTUs as response variable and modelled them with a log‐normal Poisson model. Explanatory variables included log‐transformed sequencing depth, trunk diameter at the sampling location, decay class (categorical: 2, 3 or 4), epiphyte cover on the surface of the trunk (%) and landscape setting (urban or semi‐natural). We included three levels of random effects to account for the hierarchical sampling design: samples, tree trunks and sites. In addition, we included information on phylogenetic relationships with a taxonomy‐based tree following the classifications of the UNITE database v8.2 (Abarenkov *et al*., 2020; Supplementary Table E1).

We fitted the model with two Markov Chain Monte Carlo chains, each of which consisted of 200 000 iterations, out of which we discarded the first 50 000 as burn‐in and thinned the remaining by 100 to yield a total of 3000 posterior samples. We assessed the convergence of the chains by examining the distribution of the potential scale reduction factor over the parameters that measure the responses of the OTUs to the fixed effects included in the model.

## Supporting information


**Appendix S1:** Supporting Information.

## Data Availability

High‐throughput sequence reads are available as NCBI BioSample accessions (SAMN19307225‐SAMN19307584) under BioProject PRJNA732060. OTU table including sequence read counts and sample metadata are deposited in the Dryad database https://doi.org/10.5061/dryad.9w0vt4bfr
