# Supplementary material for: Landscape context and substrate characteristics shape fungal communities of dead spruce in urban and semi‐natural forests
Source: Environ Microbiol. 2022 Jan 26;24(8):3451–62. doi: 10.1111/1462-2920.15903 (PMC9543266; doi:10.1111/1462-2920.15903)
Supplement: Supplementary file 1 — Appendix S1: Supporting Information. [file EMI-24-3451-s001.doc]

Landscape context and substrate characteristics shape fungal communities of dead spruce in urban and semi-natural forests

SUPPLEMENTARY INFORMATION

Aku Korhonen^1*^, Otto Miettinen^2^, Johan D. Kotze^3^, Leena Hamberg^1^

^1^ Natural Resources Institute Finland (Luke)

^2^ Finnish Museum of Natural History, University of Helsinki

^3^ Ecosystems and Environment Research Programme, Faculty of Biological and Environmental Sciences, University of Helsinki

*Corresponding author:

Aku Korhonen

Email: akuolavikorhonen@gmail.com

Supplement A. Sampling scheme and measurements on downed spruce trunks

Supplement B. Statistical details of the results from the ordination analysis, path analysis and joint species distribution modeling

Supplement C. Tree trunks, their locations and associated variables

Supplement D. Bioinformatics pipeline

Supplement E. Systematic taxonomy of fungal taxa applied in the joint species distribution modeling

Supplement A. Sampling scheme and measurements on downed spruce trunks


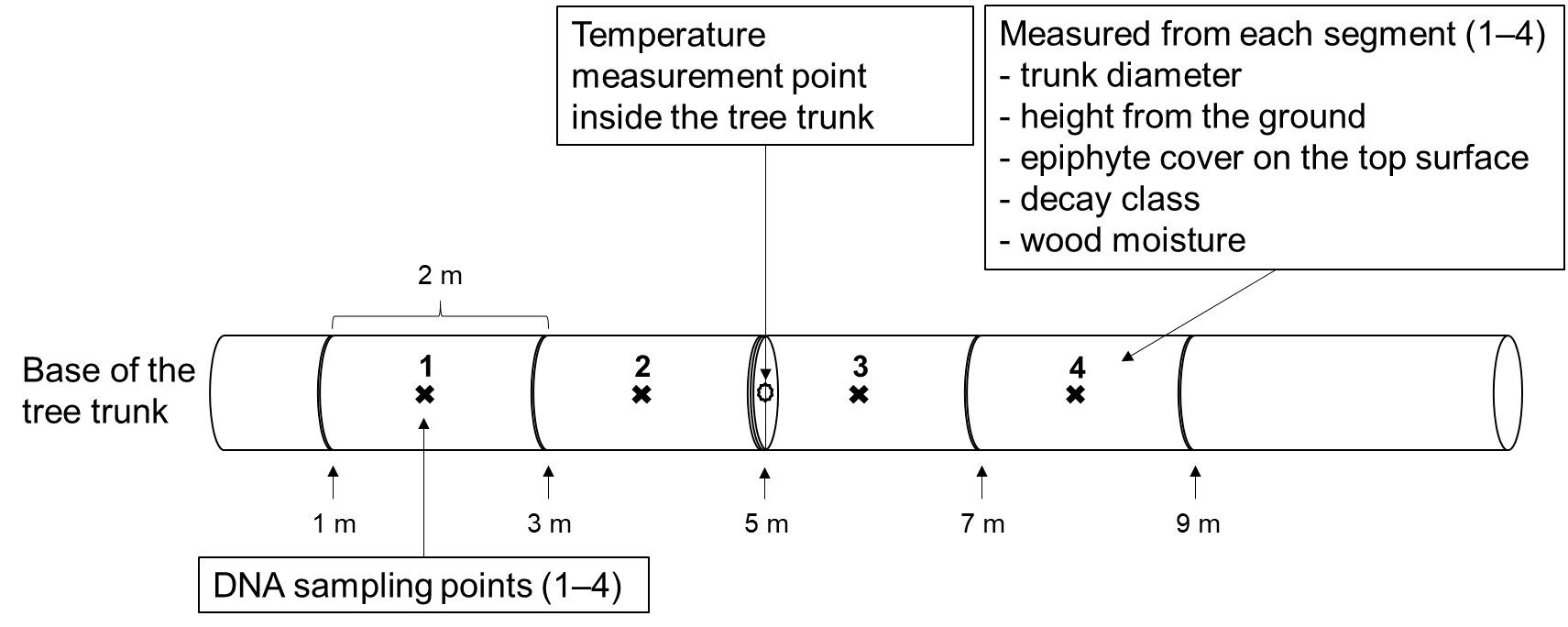


**Supplementary Fig. A1.** Data collection and DNA sampling from the tree trunks. DNA was sampled and measurements were taken between 1 and 9 m from the base of the tree trunk. Measurements were taken from 2 m long sections along the trunk, and DNA was sampled from the middle of each 2 m section. Temperature was measured at the midpoint of the trunk.

Supplement B. Statistical details of the results from the ordination analysis, path analysis and joint species distribution modeling

**Supplementary Table B1.** Relationships between environmental variables and the first two axes of the NMDS ordination of the fungal community composition. Goodness of fit is expressed as *R^2^* (variation explained by the regression of environmental variable on ordination axes). *P* values for goodness of fit are based on 10 000 permutations. Relationships with a statistical significance level *p* < 0.05 are indicated in bold, and those with 0.05 ≤ *p* < 0.10 are underlined (*n* = 90).

| Variable | NMDS1 | NMDS2 | *R*^2^ | *p* |
| --- | --- | --- | --- | --- |
| Canopy openness (%) | 0.126 | -0.992 | 0.020 | 0.420 |
| Distance from the forest edge (m) ^a^ | 0.209 | -0.978 | **0.225** | **<0.001** |
| Ground vegetation cover (%) ^b^ | -0.270 | -0.963 | 0.043 | 0.149 |
| Height from the ground (cm) | -0.012 | 1.000 | 0.013 | 0.564 |
| Trunk diameter (cm) | -0.201 | -0.980 | 0.058 | 0.070 |
| Decay class (2–4) | 0.822 | -0.569 | **0.087** | **0.019** |
| Epiphyte cover (%) | -0.949 | -0.315 | **0.253** | **<0.001** |
| Wood moisture content (%) | 0.425 | -0.905 | **0.235** | **<0.001** |
| Daily maximum 4 h temperature change (°C) ^c^ | 0.293 | 0.956 | **0.093** | **0.014** |
| Mean temperature (°C) ^c^ | 0.278 | 0.961 | 0.060 | 0.067 |
| Maximum summer temperature (°C) ^c^ | 0.245 | 0.969 | **0.230** | **<0.001** |
| Simpson’s diversity index (numeric) | 0.990 | 0.141 | **0.070** | **0.044** |
| Red-listed species (count) | 0.999 | -0.046 | **0.263** | **<0.001** |
| ^a^ Distance was log_10_-transformed.  ^b^ Measured in a 10 × 10 m square around the tree trunk.  ^c^ Measured between 1 May and 29 September, 2019. | | | | |

**Supplementary Table B2.** Statistical details of the refined path model. Component model structures are presented in the first box. Coefficients (unstandardized) with their statistical descriptives are presented in the second box. Tests of directed separation, testing whether variables that were assumed to be independent can truly be considered independent based on the data, are presented in the third box. Individual *R*^2^ values for endogenous variables are presented in the fourth box. Marginal *R*^2^ describes the variance explained by fixed effects only while conditional *R*^2^ describes variance explained by both fixed and random effects. Goodness-of-fit for the whole path model is presented in the fifth box.

| Structural Equation Model components | | | | |  |  |
| --- | --- | --- | --- | --- | --- | --- |
| Dependent variable | Explanatory variables | Random factor | Probability distribution | Link function |  |  |
| Simpson’s diversity index (SDI) | Decay class | Site | normal | - |  |  |
| Red-listed species | Epiphyte cover +  Naturalness | Site | Poisson | log |  |  |
| Epiphyte cover | Decay class + Naturalness | Site | binomial | logit |  |  |
| Wood moisture | Height + Decay class + Diameter + Naturalness | Site | normal | - |  |  |
| Daily maximum 4 h temperature change | Diameter + Wood moisture | Site | normal | - |  |  |
|  |  |  |  |  |  |  |
| Coefficients | | | | | | |
| Response | Predictor | Estimate | SE | df | Critical value | *p* |
| SDI | Decay class | 2.052 | 0.634 | 87.031 | 10.035 | 0.002 |
| Red-listed species | Epiphyte cover | -0.917 | 0.486 | 90 | -1.886 | 0.059 |
| Red-listed species | Naturalness | 0.954 | 0.321 | 90 | 2.970 | 0.003 |
| Epiphyte cover | Decay class | -0.641 | 0.347 | 90 | -1.847 | 0.065 |
| Epiphyte cover | Naturalness | 1.378 | 0.526 | 90 | 2.622 | 0.009 |
| Wood moisture | Height | -0.237 | 0.089 | 81.081 | 6.891 | 0.010 |
| Wood moisture | Diameter | 0.540 | 0.260 | 83.531 | 4.085 | 0.047 |
| Wood moisture | Decay class | 0.038 | 0.015 | 84.715 | 5.988 | 0.017 |
| Wood moisture | Naturalness | 0.076 | 0.030 | 28.810 | 6.327 | 0.018 |
| Daily maximum 4 h temperature change | Diameter | -3.626 | 0.712 | 86.854 | 24.802 | <0.001 |
| Daily maximum 4 h temperature change | Wood moisture | -0.848 | 0.268 | 86.443 | 9.615 | 0.003 |
|  |  |  |  |  |  |  |
| Tests of directed separation | | | |  |  |  |
| Independence claim | df | Critical value | *p* |  |  |  |
| SDI ~ Diameter | 73.652 | 0.001 | 0.971 |  |  |  |
| SDI ~ Height | 86.901 | 0.044 | 0.834 |  |  |  |
| SDI ~ Wood moisture | 81.454 | 0.894 | 0.347 |  |  |  |
| SDI ~ Epiphyte cover | 79.301 | 2.441 | 0.122 |  |  |  |
| SDI ~ Naturalness | 27.947 | 0.217 | 0.645 |  |  |  |
| Red-listed species ~ Diameter | 90 | 0.278 | 0.781 |  |  |  |
| Red-listed species ~ Decay class | 90 | 0.348 | 0.727 |  |  |  |
| Red-listed species ~ Height | 90 | -1.022 | 0.307 |  |  |  |
| Red-listed species ~ Wood moisture | 90 | 1.161 | 0.246 |  |  |  |
| Red-listed species  ~ Daily maximum 4 h temperature change | 90 | 0.510 | 0.610 |  |  |  |
| Daily maximum 4 h temperature change  ~ Height | 79.653 | 0.154 | 0.695 |  |  |  |
| Daily maximum 4 h temperature change  ~ Decay class | 84.112 | 0.225 | 0.636 |  |  |  |
| Daily maximum 4 h temperature change  ~ Epiphyte cover | 83.697 | 2.286 | 0.134 |  |  |  |
| Daily maximum 4 h temperature change  ~ Naturalness | 30.034 | 2.203 | 0.148 |  |  |  |
| Epiphyte cover ~ Diameter | 90 | 1.240 | 0.215 |  |  |  |
| Epiphyte cover ~ Height | 90 | -0.711 | 0.477 |  |  |  |
| Wood moisture ~ Epiphyte cover | 83.963 | 0.001 | 0.983 |  |  |  |
|  |  |  |  |  |  |  |
| Individual *R*² | | | |  |  |  |
| Response | Method | Marginal | Conditional |  |  |  |
| SDI | none | 0.11 | 0.11 |  |  |  |
| Red-listed species | trigamma | 0.03 | 0.03 |  |  |  |
| Epiphyte cover | delta | 0.12 | 0.12 |  |  |  |
| Wood moisture | none | 0.24 | 0.35 |  |  |  |
| Daily maximum 4 h temperature change | none | 0.35 | 0.51 |  |  |  |
|  |  |  |  |  |  |  |
| Global goodness-of-fit | | | | | | |
| Fisher's *C* = 32.049, df = 38, *p* = 0.74 | | | | | | |


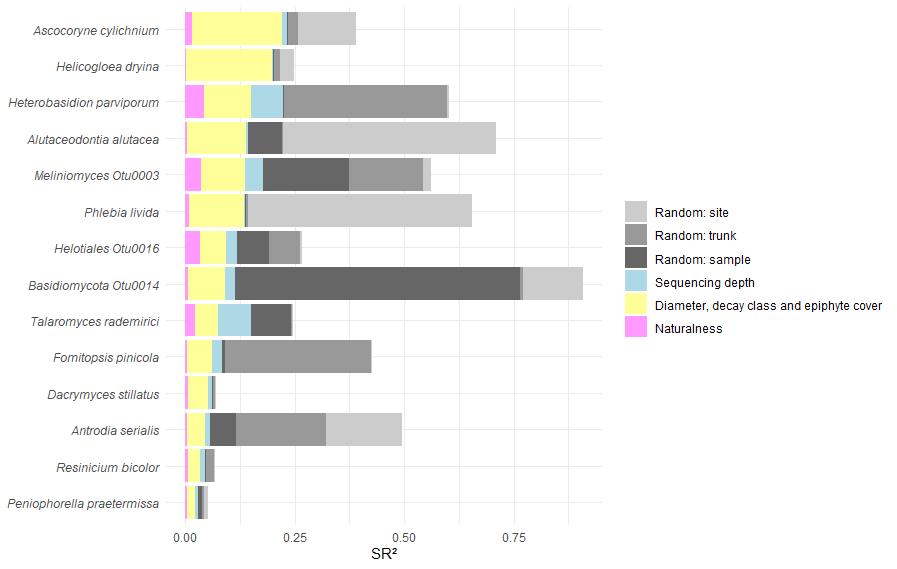


**Supplementary Fig. B1.** Variance partitioning of the joint species distribution model. Explained variance (*SR^2^*) was partitioned among grouped environmental variables (fixed effects) and random effects. Fixed effects have been grouped into substrate-level variables (diameter, decay class and epiphyte cover) and stand-level variables (Naturalness: urban or semi-natural). Total length of the bar indicates how much of variance was explained by the model for each OTU (identified to species or higher taxonomic rank) in total. Length of the coloured sections indicate how much of the explained variation is attributed to measured environmental variability (fixed effects: purple and yellow) and how much to variation in sequencing depth (blue) and unmeasured variability captured by the random effects (grey). OTUs are ordered according how much variance was explained by environmental fixed effects. In the calculation of variance partitioning, covariances among variables have been accounted for within each group but not for between groups.

Supplement C. Tree trunks, their locations and associated variables.

**Supplementary Table C1.** Tree trunks, their locations and associated variables.

| Trunk number | Site number | Site name | Municipality | X coordinate (WGS84) | Y coordinate (WGS84) | Forest category | Local volume of dead spruce (m³/ha) ^a^ | Human population density (residents per km²) ^b^ | Distance to the nearest forest edge (m) | Canopy openness (%) ^c^ | Ground vegetation cover (%) ^d^ | Height from the ground (m) ^e^ | Trunk diameter (m) ^e^ | Decay class ^e^ | Epiphyte cover (%) ^e^ | Wood moisture (%) ^e^ | Daily maximum 4 h temperature change inside the tree trunk (°C) ^f^ | Mean temperature inside the tree trunk (°C) ^f^ | Maximum summer temperature inside the tree trunk (°C) ^f^ | Simpson’s diversity index of fungal community (inverse Simpson concentration) | Number of red-listed species |
| --- | --- | --- | --- | --- | --- | --- | --- | --- | --- | --- | --- | --- | --- | --- | --- | --- | --- | --- | --- | --- | --- |
| 1 | 1 | Tonttuvuori East | Helsinki | 25.09448 | 60.16279 | urban | 57 | 32 | 16 | 17 % | 89 % | 0.00 | 0.29 | 2 | 98 % | 44 % | 0,67 | 6.99 | 22 | 1.97 | 0 |
| 2 | 1 | Tonttuvuori East | Helsinki | 25.09404 | 60.16335 | urban | 57 | 32 | 33 | 18 % | 99 % | 0.02 | 0.26 | 3 | 97 % | 49 % | 0,91 | 6.28 | 21 | 2.19 | 0 |
| 3 | 1 | Tonttuvuori East | Helsinki | 25.09387 | 60.16356 | urban | 57 | 32 | 33 | 20 % | 61 % | 0.24 | 0.35 | 3 | 12 % | 42 % | 0,68 | 6.18 | 23.5 | 7.35 | 0 |
| 4 | 2 | Hallainvuori South | Helsinki | 25.04697 | 60.22182 | urban | 15 | 732 | 8 | 36 % | 58 % | 0.10 | 0.27 | 2 | 0 % | 62 % | 1,05 | 6.07 | 23 | 6.15 | 1 |
| 5 | 2 | Hallainvuori South | Helsinki | 25.04686 | 60.22203 | urban | 15 | 732 | 30 | 19 % | 91 % | 0.22 | 0.31 | 4 | 2 % | 70 % | 0,78 | 6.34 | 24 | 2.23 | 2 |
| 6 | 3 | Hallainvuori West | Helsinki | 25.04438 | 60.22689 | urban | 25 | 3240 | 35 | 27 % | 23 % | 0.14 | 0.24 | 4 | 9 % | 42 % | 1,22 | 6.00 | 25 | 11.99 | 1 |
| 7 | 3 | Hallainvuori West | Helsinki | 25.04466 | 60.22714 | urban | 25 | 3240 | 39 | 28 % | 88 % | 0.01 | 0.27 | 3 | 16 % | 54 % | 1,08 | 6.66 | 24.5 | 3.11 | 0 |
| 8 | 4 | Oulunkylä 2024 | Helsinki | 24.94871 | 60.2351 | urban | 1 | 2979 | 18 | 23 % | 76 % | 0.30 | 0.30 | 2 | 0 % | 41 % | 1,04 | 6.04 | 24 | 5.05 | 0 |
| 9 | 5 | Länsisalmi | Vantaa | 25.15601 | 60.24805 | urban | 70 | 10 | 47 | 21 % | 93 % | 0.18 | 0.27 | 3 | 52 % | 39 % | 1,70 | 6.00 | 25.5 | 3.72 | 1 |
| 10 | 5 | Länsisalmi | Vantaa | 25.15635 | 60.24814 | urban | 70 | 10 | 46 | 16 % | 54 % | 0.25 | 0.25 | 2 | 36 % | 41 % | 1,80 | 5.70 | 24 | 4.79 | 0 |
| 11 | 6 | Vuosaari 199 | Helsinki | 25.14784 | 60.2245 | urban | 5 | 3329 | 71 | 25 % | 58 % | 0.04 | 0.25 | 4 | 0 % | 43 % | 1,37 | 5.37 | 23 | 6.67 | 0 |
| 12 | 6 | Vuosaari 199 | Helsinki | 25.14779 | 60.22417 | urban | 5 | 3329 | 56 | 23 % | 82 % | 0.17 | 0.26 | 3 | 2 % | 37 % | 1,16 | 5.92 | 22.5 | 3.45 | 0 |
| 13 | 7 | Vuosaarenhuippu North | Helsinki | 25.15706 | 60.23029 | urban | 31 | 0 | 25 | 21 % | 60 % | 0.05 | 0.31 | 3 | 0 % | 39 % | 1,13 | 6.12 | 24.5 | 5.12 | 1 |
| 14 | 7 | Vuosaarenhuippu North | Helsinki | 25.15735 | 60.23133 | urban | 31 | 0 | 60 | 20 % | 71 % | 0.29 | 0.29 | 4 | 49 % | 28 % | 1,13 | 5.97 | 23.5 | 21.36 | 0 |
| 15 | 7 | Vuosaarenhuippu North | Helsinki | 25.15773 | 60.23215 | urban | 31 | 0 | 24 | 24 % | 91 % | 0.00 | 0.25 | 3 | 96 % | 43 % | 0,89 | 5.52 | 21 | 2.77 | 0 |
| 16 | 8 | Uutela | Helsinki | 25.16462 | 60.20036 | urban | 22 | 159 | 155 | 14 % | 87 % | 0.17 | 0.26 | 2 | 78 % | 35 % | 1,23 | 5.92 | 24.5 | 3.18 | 0 |
| 17 | 8 | Uutela | Helsinki | 25.16749 | 60.19921 | urban | 22 | 159 | 182 | 20 % | 73 % | 0.26 | 0.24 | 2 | 6 % | 34 % | 1,39 | 5.44 | 23.5 | 5.12 | 0 |
| 18 | 9 | Gråbergsbacken | Espoo | 24.61889 | 60.1764 | urban | 26 | 1154 | 40 | 21 % | 84 % | 0.03 | 0.37 | 3 | 100 % | 58 % | 0,61 | 5.71 | 21 | 3.64 | 0 |
| 19 | 9 | Gråbergsbacken | Espoo | 24.61923 | 60.1765 | urban | 26 | 1154 | 53 | 26 % | 69 % | 0.04 | 0.33 | 4 | 26 % | 36 % | 1,02 | 6.72 | 26.5 | 5.01 | 0 |
| 20 | 9 | Gråbergsbacken | Espoo | 24.61997 | 60.17687 | urban | 26 | 1154 | 45 | 19 % | 43 % | 0.10 | 0.35 | 2 | 8 % | 37 % | 0,86 | 6.34 | 24.5 | 3.83 | 0 |
| 21 | 9 | Gråbergsbacken | Espoo | 24.61926 | 60.17713 | urban | 26 | 1154 | 25 | 19 % | 81 % | 0.12 | 0.35 | 3 | 100 % | 45 % | 0,91 | 5.30 | 22.5 | 2.88 | 0 |
| 22 | 10 | Petikonmäki | Vantaa | 24.80845 | 60.28033 | urban | 20 | 1 | 48 | 22 % | 40 % | 0.23 | 0.38 | 4 | 10 % | 39 % | 0,63 | 5.73 | 23 | 5.62 | 0 |
| 23 | 10 | Petikonmäki | Vantaa | 24.80914 | 60.2798 | urban | 20 | 1 | 22 | 24 % | 78 % | 0.31 | 0.24 | 3 | 6 % | 39 % | 0,99 | 5.36 | 24.5 | 18.84 | 0 |
| 24 | 10 | Petikonmäki | Vantaa | 24.8091 | 60.27893 | urban | 20 | 1 | 25 | 22 % | 84 % | 0.18 | 0.24 | 2 | 3 % | 23 % | 1,44 | 5.77 | 26 | 3.46 | 1 |
| 25 | 10 | Petikonmäki | Vantaa | 24.80552 | 60.27839 | urban | 20 | 1 | 78 | 33 % | 88 % | 0.22 | 0.29 | 3 | 28 % | 39 % | 1,50 | 5.94 | 25.5 | 9.64 | 0 |
| 26 | 11 | Soltorp | Vantaa | 24.79002 | 60.26425 | urban | 94 | 154 | 93 | 22 % | 63 % | 0.06 | 0.43 | 4 | 0 % | 49 % | 0,80 | 6.26 | 23 | 4.75 | 0 |
| 27 | 11 | Soltorp | Vantaa | 24.7898 | 60.26405 | urban | 94 | 154 | 71 | 24 % | 84 % | 0.33 | 0.39 | 2 | 50 % | 34 % | 1,12 | 5.94 | 24 | 10.60 | 0 |
| 28 | 11 | Soltorp | Vantaa | 24.78948 | 60.26362 | urban | 94 | 154 | 62 | 23 % | 87 % | 0.12 | 0.28 | 3 | 74 % | 22 % | 1,38 | 5.43 | 24 | 5.12 | 0 |
| 29 | 11 | Soltorp | Vantaa | 24.79145 | 60.26383 | urban | 94 | 154 | 53 | 34 % | 57 % | 0.00 | 0.34 | 3 | 43 % | 58 % | 0,83 | 6.53 | 22.5 | 2.88 | 0 |
| 30 | 12 | Vaskivuori | Vantaa | 24.86598 | 60.26215 | urban | 19 | 4349 | 99 | 27 % | 3 % | 0.01 | 0.28 | 3 | 0 % | 32 % | 2,40 | 7.28 | 28 | 3.08 | 1 |
| 31 | 12 | Vaskivuori | Vantaa | 24.86499 | 60.26217 | urban | 19 | 4349 | 150 | 21 % | 54 % | 0.01 | 0.33 | 3 | 0 % | 46 % | 0,96 | 7.06 | 24 | 2.25 | 1 |
| 32 | 12 | Vaskivuori | Vantaa | 24.86489 | 60.26345 | urban | 19 | 4349 | 137 | 20 % | 60 % | 0.01 | 0.24 | 4 | 0 % | 45 % | 1,34 | 6.27 | 24.5 | 16.39 | 0 |
| 33 | 12 | Vaskivuori | Vantaa | 24.86603 | 60.26388 | urban | 19 | 4349 | 60 | 21 % | 24 % | 0.10 | 0.31 | 3 | 6 % | 33 % | 0,97 | 5.51 | 23.5 | 14.86 | 0 |
| 34 | 13 | Haltiavuori | Helsinki | 24.90875 | 60.26218 | urban | 19 | 0 | 368 | 21 % | 66 % | 0.03 | 0.33 | 4 | 16 % | 67 % | 0,76 | 6.74 | 24 | 11.87 | 2 |
| 35 | 13 | Haltiavuori | Helsinki | 24.90741 | 60.26263 | urban | 19 | 0 | 301 | 18 % | 83 % | 0.07 | 0.25 | 4 | 0 % | 37 % | 1,50 | 6.30 | 25.5 | 8.36 | 2 |
| 36 | 13 | Haltiavuori | Helsinki | 24.90619 | 60.26235 | urban | 19 | 0 | 228 | 22 % | 100 % | 0.10 | 0.27 | 3 | 3 % | 42 % | 1,64 | 5.97 | 24 | 4.39 | 0 |
| 37 | 14 | Jyrängönpuisto | Helsinki | 24.9561 | 60.20749 | urban | 24 | 4234 | 64 | 31 % | 26 % | 0.10 | 0.34 | 3 | 0 % | 40 % | 0,83 | 6.13 | 22.5 | 3.10 | 0 |
| 38 | 15 | Vierumäki | Vantaa | 25.04087 | 60.36176 | urban | 8 | 436 | 119 | 23 % | 98 % | 0.21 | 0.26 | 2 | 3 % | 45 % | 1,53 | 5.08 | 24 | 6.04 | 1 |
| 39 | 16 | Haltiala | Helsinki | 24.92729 | 60.2717 | urban | 65 | 3 | 35 | 26 % | 98 % | 0.12 | 0.27 | 4 | 8 % | 41 % | 1,82 | 5.91 | 26 | 8.35 | 0 |
| 40 | 16 | Haltiala | Helsinki | 24.92772 | 60.27178 | urban | 65 | 3 | 8 | 30 % | 95 % | 0.19 | 0.39 | 4 | 5 % | 41 % | 0,64 | 5.67 | 22.5 | 12.23 | 0 |
| 41 | 16 | Haltiala | Helsinki | 24.92848 | 60.27104 | urban | 65 | 3 | 74 | 36 % | 95 % | 0.01 | 0.33 | 4 | 2 % | 67 % | 1,15 | 5.65 | 23.5 | 17.74 | 0 |
| 42 | 17 | Pakilanmetsä | Helsinki | 24.91097 | 60.2488 | urban | 9 | 1643 | 50 | 25 % | 92 % | 0.21 | 0.31 | 3 | 1 % | 33 % | 1,13 | 5.87 | 24 | 12.13 | 0 |
| 43 | 17 | Pakilanmetsä | Helsinki | 24.91216 | 60.24839 | urban | 9 | 1643 | 93 | 23 % | 93 % | 0.13 | 0.29 | 2 | 57 % | 36 % | 1,54 | 5.95 | 25 | 8.48 | 0 |
| 44 | 17 | Pakilanmetsä | Helsinki | 24.91491 | 60.24856 | urban | 9 | 1643 | 62 | 18 % | 10 % | 0.04 | 0.23 | 2 | 0 % | 37 % | 1,65 | 6.06 | 25 | 2.88 | 1 |
| 45 | 17 | Pakilanmetsä | Helsinki | 24.91554 | 60.24888 | urban | 9 | 1643 | 7 | 19 % | 95 % | 0.25 | 0.25 | 4 | 31 % | 40 % | 1,30 | 5.80 | 24 | 2.21 | 0 |
| 46 | 18 | Maunulanpuisto | Helsinki | 24.92248 | 60.22453 | urban | 28 | 398 | 41 | 22 % | 45 % | 0.52 | 0.33 | 3 | 1 % | 38 % | 0,98 | 5.76 | 23.5 | 12.05 | 0 |
| 47 | 18 | Maunulanpuisto | Helsinki | 24.92193 | 60.22414 | urban | 28 | 398 | 77 | 20 % | 21 % | 0.22 | 0.27 | 3 | 20 % | 28 % | 1,14 | 6.37 | 25 | 3.70 | 1 |
| 48 | 19 | Männikkötie | Helsinki | 24.92947 | 60.22396 | urban | 11 | 4932 | 28 | 23 % | 44 % | 0.03 | 0.26 | 3 | 3 % | 25 % | 1,92 | 5.95 | 25 | 7.72 | 0 |
| 49 | 19 | Männikkötie | Helsinki | 24.92943 | 60.22369 | urban | 11 | 4932 | 30 | 18 % | 64 % | 0.16 | 0.28 | 3 | 0 % | 41 % | 1,16 | 6.09 | 24.5 | 3.28 | 1 |
| 50 | 20 | Ilmala North | Helsinki | 24.91233 | 60.20829 | urban | 27 | 668 | 18 | 20 % | 80 % | 0.06 | 0.29 | 4 | 0 % | 53 % | 0,69 | 5.75 | 22.5 | 18.46 | 1 |
| 51 | 20 | Ilmala North | Helsinki | 24.91234 | 60.20792 | urban | 27 | 668 | 53 | 27 % | 79 % | 0.21 | 0.26 | 3 | 2 % | 45 % | 1,36 | 5.92 | 23.5 | 6.66 | 1 |
| 52 | 20 | Ilmala South | Helsinki | 24.91312 | 60.2063 | urban | 28 | 668 | 33 | 28 % | 88 % | 0.08 | 0.32 | 2 | 3 % | 52 % | 0,65 | 6.40 | 21.5 | 1.57 | 0 |
| 53 | 22 | Länsimäki | Vantaa | 25.12761 | 60.23875 | urban | 33 | 1164 | 51 | 21 % | 55 % | 0.24 | 0.32 | 3 | 0 % | 31 % | 1,08 | 6.66 | 26.5 | 3.77 | 0 |
| 54 | 22 | Länsimäki | Vantaa | 25.1241 | 60.23947 | urban | 33 | 1164 | 64 | 19 % | 81 % | 0.18 | 0.32 | 3 | 0 % | 44 % | 1,03 | 5.94 | 24.5 | 3.95 | 0 |
| 55 | 22 | Länsimäki | Vantaa | 25.12646 | 60.23972 | urban | 33 | 1164 | 121 | 16 % | 83 % | 0.00 | 0.29 | 4 | 7 % | 62 % | 0,59 | 5.86 | 21 | 3.43 | 1 |
| 56 | 22 | Länsimäki | Vantaa | 25.1263 | 60.24082 | urban | 33 | 1164 | 61 | 23 % | 61 % | 0.38 | 0.27 | 3 | 2 % | 40 % | 1,48 | 5.40 | 24 | 4.34 | 1 |
| 57 | 23 | Meri-Rastila | Helsinki | 25.10667 | 60.19752 | urban | 9 | 1008 | 146 | 18 % | 98 % | 0.22 | 0.27 | 3 | 0 % | 40 % | 1,00 | 5.88 | 24.5 | 3.46 | 1 |
| 58 | 23 | Meri-Rastila | Helsinki | 25.10666 | 60.19821 | urban | 9 | 1008 | 185 | 22 % | 78 % | 0.05 | 0.27 | 3 | 2 % | 43 % | 1,26 | 6.53 | 26 | 3.77 | 0 |
| 59 | 23 | Meri-Rastila | Helsinki | 25.10642 | 60.19772 | urban | 9 | 1008 | 171 | 21 % | 88 % | 0.04 | 0.27 | 2 | 100 % | 37 % | 1,08 | 5.56 | 23 | 4.69 | 1 |
| 60 | 23 | Meri-Rastila | Helsinki | 25.10613 | 60.2016 | urban | 9 | 1008 | 22 | 16 % | 94 % | 0.03 | 0.25 | 2 | 1 % | 41 % | 1,19 | 6.48 | 25.5 | 13.46 | 0 |
| 61 | 24 | Kivinokka | Helsinki | 25.01704 | 60.19661 | urban | 51 | 2376 | 164 | 16 % | 91 % | 0.09 | 0.27 | 4 | 49 % | 63 % | 1,10 | 5.98 | 23 | 3.71 | 0 |
| 62 | 24 | Kivinokka | Helsinki | 25.01662 | 60.19741 | urban | 51 | 2376 | 137 | 24 % | 97 % | 0.26 | 0.34 | 3 | 84 % | 36 % | 0,96 | 5.61 | 22.5 | 3.21 | 1 |
| 63 | 24 | Kivinokka | Helsinki | 25.01719 | 60.19819 | urban | 51 | 2376 | 49 | 29 % | 88 % | 0.34 | 0.31 | 3 | 81 % | 29 % | 0,95 | 5.43 | 23.5 | 10.42 | 0 |
| 64 | 24 | Kivinokka | Helsinki | 25.02004 | 60.19701 | urban | 51 | 2376 | 55 | 25 % | 51 % | 0.13 | 0.35 | 4 | 0 % | 77 % | 0,74 | 6.19 | 23 | 17.81 | 1 |
| 65 | 25 | Mölylä | Helsinki | 25.02305 | 60.21241 | urban | 50 | 56 | 27 | 22 % | 95 % | 0.13 | 0.35 | 2 | 4 % | 56 % | 0,85 | 5.92 | 24 | 3.30 | 0 |
| 66 | 25 | Mölylä | Helsinki | 25.02206 | 60.21219 | urban | 50 | 56 | 84 | 34 % | 94 % | 0.04 | 0.36 | 2 | 68 % | 43 % | 0,73 | 5.67 | 21.5 | 1.74 | 0 |
| 67 | 26 | Finnträsk | Kirkkonummi | 24.55526 | 60.13437 | semi-natural | 25 | 0 | 39 | 23 % | 98 % | 0.25 | 0.31 | 4 | 18 % | 51 % | 1,13 | 5.45 | 23 | 6.18 | 0 |
| 68 | 26 | Finnträsk | Kirkkonummi | 24.55584 | 60.13412 | semi-natural | 25 | 0 | 79 | 19 % | 96 % | 0.36 | 0.36 | 2 | 100 % | 36 % | 0,81 | 5.74 | 24 | 8.47 | 0 |
| 69 | 26 | Finnträsk | Kirkkonummi | 24.55623 | 60.13548 | semi-natural | 25 | 0 | 82 | 28 % | 91 % | 0.24 | 0.32 | 4 | 87 % | 45 % | 0,91 | 5.49 | 23.5 | 4.49 | 0 |
| 70 | 27 | Mustametsä | Mäntsälä | 25.15263 | 60.62373 | semi-natural | 59 | 0 | 104 | 21 % | 94 % | 0.12 | 0.26 | 3 | 100 % | 54 % | 1,15 | 4.85 | 21 | 7.21 | 0 |
| 71 | 27 | Mustametsä | Mäntsälä | 25.15202 | 60.62364 | semi-natural | 59 | 0 | 100 | 31 % | 90 % | 0.03 | 0.28 | 3 | 15 % | 47 % | 0,50 | 5.60 | 19.5 | 9.33 | 0 |
| 72 | 27 | Mustametsä | Mäntsälä | 25.15173 | 60.62332 | semi-natural | 59 | 0 | 126 | 27 % | 92 % | 0.24 | 0.29 | 4 | 98 % | 57 % | 1,23 | 4.70 | 23 | 3.54 | 0 |
| 73 | 28 | Metsäkulma | Mäntsälä | 25.41132 | 60.64211 | semi-natural | 50 | 0 | 201 | 20 % | 94 % | 0.00 | 0.32 | 4 | 2 % | 40 % | 1,32 | 6.13 | 23 | 20.83 | 3 |
| 74 | 28 | Metsäkulma | Mäntsälä | 25.41155 | 60.64289 | semi-natural | 50 | 0 | 329 | 25 % | 89 % | 0.55 | 0.26 | 3 | 43 % | 41 % | 0,96 | 4.73 | 20.5 | 2.91 | 1 |
| 75 | 28 | Metsäkulma | Mäntsälä | 25.41135 | 60.64328 | semi-natural | 50 | 0 | 355 | 25 % | 98 % | 0.02 | 0.33 | 3 | 64 % | 64 % | 0,70 | 5.61 | 21.5 | 11.13 | 1 |
| 76 | 29 | Rörstrand | Sipoo | 25.19343 | 60.45861 | semi-natural | 34 | 0 | 473 | 35 % | 93 % | 0.08 | 0.28 | 2 | 97 % | 51 % | 1,09 | 5.87 | 23.5 | 4.56 | 0 |
| 77 | 29 | Rörstrand | Sipoo | 25.19274 | 60.45826 | semi-natural | 34 | 0 | 500 | 34 % | 96 % | 0.20 | 0.33 | 4 | 21 % | 37 % | 0,91 | 5.73 | 25 | 10.95 | 1 |
| 78 | 29 | Rörstrand | Sipoo | 25.19168 | 60.45955 | semi-natural | 34 | 0 | 589 | 30 % | 95 % | 0.11 | 0.32 | 2 | 45 % | 47 % | 0,87 | 5.91 | 21 | 12.48 | 3 |
| 79 | 30 | Myllypuron kanjoni | Vihti | 24.50387 | 60.33166 | semi-natural | 64 | 0 | 369 | 16 % | 98 % | 0.25 | 0.33 | 2 | 0 % | 53 % | 1,23 | 5.20 | 22.5 | 3.07 | 2 |
| 80 | 30 | Myllypuron kanjoni | Vihti | 24.50376 | 60.33199 | semi-natural | 64 | 0 | 403 | 17 % | 94 % | 0.08 | 0.37 | 2 | 92 % | 39 % | 1,29 | 5.26 | 23 | 5.01 | 1 |
| 81 | 30 | Myllypuron kanjoni | Vihti | 24.50282 | 60.33274 | semi-natural | 64 | 0 | 500 | 27 % | 97 % | 0.14 | 0.30 | 2 | 10 % | 32 % | 2,03 | 6.17 | 26.5 | 2.54 | 0 |
| 82 | 31 | Kummelbergen | Sipoo | 25.24031 | 60.47769 | semi-natural | 35 | 0 | 189 | 25 % | 95 % | 0.00 | 0.30 | 3 | 51 % | 59 % | 0,83 | 5.58 | 20 | 10.31 | 1 |
| 83 | 31 | Kummelbergen | Sipoo | 25.24064 | 60.47749 | semi-natural | 35 | 0 | 208 | 24 % | 97 % | 0.06 | 0.42 | 3 | 85 % | 53 % | 0,61 | 5.53 | 21.5 | 6.66 | 1 |
| 84 | 31 | Kummelbergen | Sipoo | 25.24089 | 60.47743 | semi-natural | 35 | 0 | 224 | 32 % | 98 % | 0.60 | 0.32 | 3 | 55 % | 59 % | 1,26 | 4.69 | 22.5 | 4.85 | 0 |
| 85 | 32 | Poukamoinmäki | Karkkila | 24.40218 | 60.58579 | semi-natural | 45 | 2 | 161 | 16 % | 99 % | 0.00 | 0.38 | 2 | 100 % | 77 % | 0,87 | 4.03 | 20 | 4.52 | 1 |
| 86 | 32 | Poukamoinmäki | Karkkila | 24.40125 | 60.58585 | semi-natural | 45 | 2 | 141 | 24 % | 93 % | 0.09 | 0.31 | 4 | 11 % | 77 % | 1,26 | 4.71 | 22.5 | 4.67 | 0 |
| 87 | 32 | Poukamoinmäki | Karkkila | 24.40049 | 60.58626 | semi-natural | 45 | 2 | 121 | 28 % | 96 % | 0.23 | 0.43 | 3 | 83 % | 64 % | 1,18 | 4.66 | 22 | 4.76 | 2 |
| 88 | 33 | Herukkapuro | Vantaa | 24.7756 | 60.32753 | semi-natural | 79 | 34 | 419 | 23 % | 98 % | 0.06 | 0.35 | 2 | 9 % | 37 % | 1,10 | 6.27 | 23.5 | 5.91 | 0 |
| 89 | 33 | Herukkapuro | Vantaa | 24.77518 | 60.32775 | semi-natural | 79 | 34 | 402 | 24 % | 97 % | 0.22 | 0.35 | 3 | 1 % | 60 % | 1,01 | 5.95 | 24.5 | 4.20 | 1 |
| 90 | 33 | Herukkapuro | Vantaa | 24.77512 | 60.32828 | semi-natural | 79 | 34 | 349 | 25 % | 65 % | 0.16 | 0.28 | 2 | 0 % | 40 % | 1,69 | 5.80 | 25 | 6.97 | 1 |
| **Urban median (mean) values** | | | | | | | **26** | **732** | **53** | **22 %** | **81 %** | **0.12** | **0.29** | **3** | **6 %** | **41 %** | **1.08** | **5.95** | **24.0** | **4.72** | **(0.38)** |
| **Semi-natural median (mean) values** | | | | | | | **48** | **0** | **216** | **25 %** | **95 %** | **0.13** | **0.32** | **3** | **48 %** | **51 %** | **1.09** | **5.56** | **22.3** | **5.46** | **(0.79)** |
| ^a^ Coarse woody debris of Norway spruce (diameter ≥ 15 cm, decay classes 2–4). Measurements from Korhonen et al., 2021, Landsc Urban Plan 215: 104222.  ^b^ Data from population grid data (1 × 1 km grid map) from year 2018 (Statistics Finland, 2020).  ^c^ Measured as the proportion of visible sky in a photo taken towards the sky from the surface of the downed trunk using a 180° lens.  ^d^ Measured in a 10 × 10 m square around the tree trunk.  ^e^ Average between 3 and 7 m from the base of the trunk.  ^f^ Measured between 1 May and 29 September 2019. | | | | | | | | | | | | | | | | | | | | | |

**Supplementary Table C2.** Pairwise correlations between variables characterizing decaying spruce trunks. Kendall's rank correlation coefficients (Kendall’s Τ) are presented in the bottom triangle and associated *p* values in the top triangle. Correlations with statistical significance level *p* < 0.05 are indicated in bold and those with 0.05 ≤ *p* < 0.10 are underlined (*n* = 90).

|  | Variable | Canopy openness (%) | Distance from the forest edge (m) ^a^ | Ground vegetation cover | Height from the ground (cm) | Trunk diameter (m) | Decay class | Epiphyte cover (%) | Wood moisture content (%) | Daily maximum 4 h temperature change (°C) ^b^ | Mean temperature (°C) ^b^ | Max. summer temperature (°C) ^b^ | Simpson’s diversity index (numeric) | Red-listed species (count) |
| --- | --- | --- | --- | --- | --- | --- | --- | --- | --- | --- | --- | --- | --- | --- |
| Environment | Canopy openness above the tree trunk (%) |  | 0.443 | 0.508 | 0.663 | 0.112 | 0.389 | 0.486 | 0.345 | 0.565 | 0.208 | 0.274 | 0.092 | 0.275 |
|  | Distance from the forest edge (m) ^a^ | 0.06 |  | **0.001** | 0.494 | 0.206 | 0.458 | 0.174 | 0.399 | 0.176 | 0.088 | 0.381 | 0.528 | **0.002** |
|  | Ground vegetation cover around the trunk | 0.05 | **0.23** |  | 0.712 | 0.062 | 0.357 | **<0.001** | **0.006** | 0.906 | **<0.001** | **0.007** | 0.720 | 0.644 |
| Structural characteristics | Height from the ground (m) | 0.03 | -0.05 | 0.03 |  | 0.939 | 0.725 | 0.875 | **0.004** | 0.130 | **0.001** | 0.095 | 0.508 | 0.483 |
|  | Trunk diameter (m) | 0.11 | 0.09 | 0.13 | 0.01 |  | 0.846 | 0.123 | **0.033** | **<0.001** | 0.717 | **0.003** | 0.579 | 0.967 |
|  | Decay class | 0.07 | -0.06 | -0.08 | -0.03 | 0.02 |  | 0.21 | **0.028** | 0.281 | 0.840 | 0.780 | **0.020** | 0.865 |
|  | Epiphyte cover on the tree trunk (%) | 0.05 | 0.10 | **0.29** | 0.01 | 0.11 | -0.11 |  | 0.464 | 0.060 | **<0.001** | **<0.001** | 0.276 | 0.061 |
| Moisture and temperature conditions | Wood moisture content (%) | 0.07 | 0.06 | **0.20** | **-0.21** | **0.15** | **0.18** | 0.05 |  | **<0.001** | 0.182 | **<0.001** | 0.490 | 0.100 |
|  | Daily maximum 4 h temperature change (°C) ^b^ | -0.04 | 0.10 | -0.01 | 0.11 | **-0.40** | -0.09 | -0.14 | **-0.26** |  | 0.799 | **<0.001** | 0.553 | 0.758 |
|  | Mean temperature inside the trunk (°C) ^b^ | -0.09 | -0.12 | **-0.26** | **-0.24** | -0.04 | 0.02 | **-0.28** | -0.10 | -0.02 |  | **<0.001** | 0.183 | 0.990 |
|  | Max. summer temperature inside the trunk (°C) ^b^ | -0.08 | -0.07 | **-0.20** | 0.13 | **-0.22** | -0.02 | **-0.29** | **-0.38** | **0.43** | **0.34** |  | 0.512 | 0.902 |
| Fungal community | Simpson’s diversity index (numeric) | 0.12 | 0.05 | -0.03 | 0.05 | 0.04 | **0.19** | -0.08 | -0.05 | 0.04 | -0.10 | 0.05 |  | 0.789 |
|  | Red-listed species (count) | -0.09 | **0.26** | 0.04 | -0.06 | <0.01 | 0.02 | -0.16 | 0.14 | 0.03 | >-0.01 | -0.01 | 0.02 |  |
| ^a^ Distance was log_10_-transformed.  ^b^ Measured between 1 May and 29 September 2019. | | | | | | | | | | | | | | |


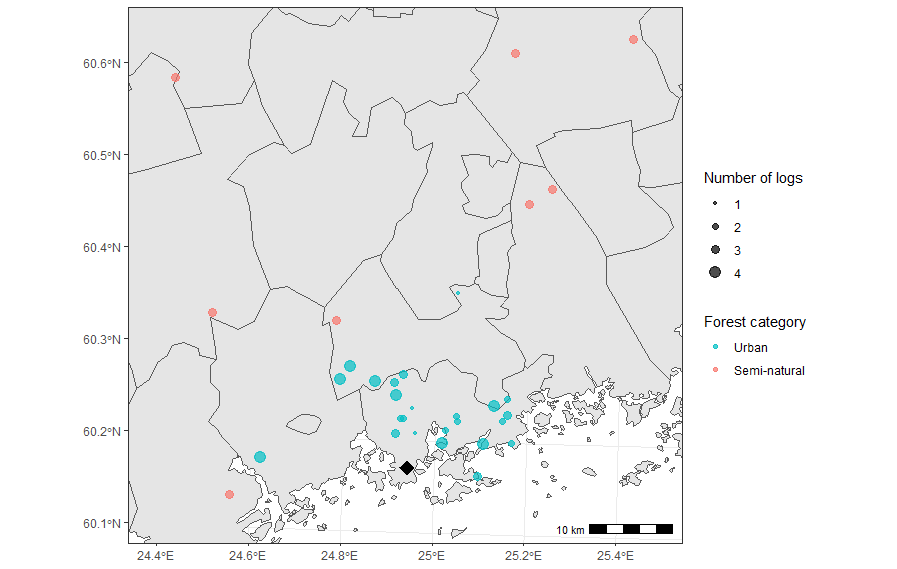


**Supplementary Fig. C1.** Locations of studied forest stands in southern Finland. Size of the dot reflects the number tree trunks sampled in the stand. Location of the Helsinki’s city center is indicated with the black diamond.

Supplement D. Bioinformatics pipeline

Quality filtering and the removal of artefacts, primer‐dimers and primers from raw sequence reads (NCBI BioSample accessions SAMN19307225-SAMN19307584 under BioProject PRJNA732060) were conducted with the PipeCraft 1.0 pipeline (Anslan *et al.*, 2017). Raw ITS sequence reads were processed according to the manual with the following specifications. Assembly of paired-end reads and initial quality ﬁltering was conducted with vsearch v1.11.1 (Rognes *et al.*, 2016) with the following parameters: minimum overlap 20, max differences 99, minimum length 150 bp, e_max 1, max ambiguous 0, and trunc qual 20. Chimera ﬁltering was performed for the reoriented reads using reference-based ﬁltering with Unite (Abarenkov *et al.*, 2010) ITS2 ref. v7.1 as the database, also removing primers and primer artifacts from sequences at this step. In addition, the fungal ITS2 region was extracted from reads with ITSx (Bengtsson‐Palme *et al.*, 2013).

The remaining 7.876 M sequences were then clustered with Swarm v2 algorithm (d = 1, fastidious = TRUE; Mahé *et al.*, 2015). These raw operational taxonomic units (OTUs) were identified to taxonomic groups with the Naïve Bayesian classifier (Wang *et al.*, 2007) using UNITE database v8.2 (Abarenkov *et al.*, 2020) as the reference database and matched to the closest representative species hypothesis in the database using BLASTn. Raw OTUs that were assigned to the same named species with at least 90% confidence were then aggregated to combined OTUs. Raw OTUs that were not assigned to any named species-level taxon were compared to others that had the same UNITE species hypothesis as their closest match; if the representative sequence of an OTU had at least 97.5% sequence similarity to any other OTU in the same group, they were merged. Raw OTUs with < 97.5% similarity to any other OTU were retained as separate OTUs.

**References**

Abarenkov, K., Nilsson, R. H., Larsson, K.‐H., Alexander, I. J., Eberhardt, U., Erland, S. *et al.* (2010) The UNITE database for molecular identification of fungi ‐ recent updates and future perspectives. New Phytol 186: 281–285.

Abarenkov, K., Zirk, A., Piirmann, T., Pöhönen, R., Ivanov, F., Nilsson, R. H., and Kõljalg, U. (2020) UNITE general FASTA release for Fungi. Version 04.02.2020 [dataset]. UNITE Community.

Anslan, S., Bahram, M., Hiiesalu, I., and Tedersoo, L. (2017) PipeCraft: flexible open‐source toolkit for bioinformatics analysis of custom high‐throughput amplicon sequencing data. Mol Ecol Resour 17: 234–240.

Bengtsson-Palme, J., Ryberg, M., Hartmann, M., Branco, S., Wang, Z., Godhe, A. *et al.* (2013) Improved software detection and extraction of ITS1 and ITS 2 from ribosomal ITS sequences of fungi and other eukaryotes for analysis of environmental sequencing data. Methods in Ecol Evol 4: 914–919.

Mahé, F., Rognes, T., Quince, C., de Vargas, C., and Dunthorn, M. (2015). Swarm v2: highly-scalable and high-resolution amplicon clustering. PeerJ 3: e1420.

Rognes, T., Flouri, T., Nichols, B., Quince, C., and Mahé, F. (2016) VSEARCH: a versatile opensource tool for metagenomics. PeerJ 4: e2584.

Wang, Q., Garrity, G. M., Tiedje, J. M., and Cole, J. R. (2007). Naïve Bayesian classifier for rapid assignment of rRNA sequences into the new bacterial taxonomy. Appl Environ Microbiol 73: 5261–5267.

Supplement E. Systematic taxonomy of fungal taxa applied in the joint species distribution modeling

**Supplementary Table E1.** Systematic taxonomy of fungal taxa applied in the joint species distribution modeling, following the taxonomy of UNITE database v8.2.

| Species (OTU) | Genus | Family | Order | Class | Phylum |
| --- | --- | --- | --- | --- | --- |
| *Talaromyces rademirici* | *Talaromyces* | Trichocomaceae | Eurotiales | Eurotiomycetes | Ascomycota |
| *Ascocoryne cylichnium* | *Ascocoryne* | Helotiaceae | Helotiales | Leotiomycetes | Ascomycota |
| *Meliniomyces* Otu0003 | *Meliniomyces* | Helotiaceae | Helotiales | Leotiomycetes | Ascomycota |
| Helotiales Otu0016 | Helotiales Otu0016 | Helotiales Otu0016 | Helotiales | Leotiomycetes | Ascomycota |
| *Peniophorella praetermissa* | *Peniophorella* | Hymenochaetales Incertae sedis 1 | Hymenochaetales | Agaricomycetes | Basidiomycota |
| *Resinicium bicolor* | *Resinicium* | Hymenochaetales Incertae sedis 2 | Hymenochaetales | Agaricomycetes | Basidiomycota |
| *Alutaceodontia alutacea* | *Alutaceodontia* | Schizoporaceae | Hymenochaetales | Agaricomycetes | Basidiomycota |
| *Antrodia serialis* | *Antrodia* | Fomitopsidaceae | Polyporales | Agaricomycetes | Basidiomycota |
| *Fomitopsis pinicola* | *Fomitopsis* | Fomitopsidaceae | Polyporales | Agaricomycetes | Basidiomycota |
| *Phlebia livida* | *Phlebia* | Meruliaceae | Polyporales | Agaricomycetes | Basidiomycota |
| *Heterobasidion parviporum* | *Heterobasidion* | Bondarzewiaceae | Russulales | Agaricomycetes | Basidiomycota |
| *Helicogloea dryina* | *Helicogloea* | Atractiellales Incertae sedis | Atractiellales | Atractiellomycetes | Basidiomycota |
| *Dacrymyces stillatus* | *Dacrymyces* | Dacrymycetaceae | Dacrymycetales | Dacrymycetes | Basidiomycota |
| Basidiomycota Otu0014 | Basidiomycota Otu0014 | Basidiomycota Otu0014 | Basidiomycota Otu0014 | Basidiomycota Otu0014 | Basidiomycota |
